# Analysis of the total serum IgE levels in patients with acute exacerbations chronic obstructive pulmonary disease: A retrospective study

**DOI:** 10.1097/MD.0000000000037792

**Published:** 2024-04-19

**Authors:** Xiang Xie, Juan Zheng, Zhen Li, Jun Qi, Lili Li, Lindong Yuan, Tingting Jiang, Ziyun Yang, Shan Qin, Xiufen Tian, Yan Wang, Peige Zhao

**Affiliations:** a Department of Respiratory and Critical Care Medicine, Liaocheng People’s Hospital, Liaocheng, China; b Joint Laboratory for Translational Medicine Research, Liaocheng People’s Hospital, Liaocheng, China; c Department of Clinical Laboratory, Liaocheng People’s Hospital, Liaocheng, China; d Department of Respiratory Medicine, Liaocheng People’s Hospital, Liaocheng, China; e Department of Endodontics, Liaocheng People’s Hospital, Liaocheng, China; f Department of Respiratory and Critical Care Medicine, Affiliated Hospital of Qingdao University, Qingdao, China.

**Keywords:** acute exacerbations chronic obstructive pulmonary disease, glucocorticoid, hospital stays, total IgE

## Abstract

Currently, few studies have demonstrated the relationship between total serum IgE (T-IgE) and acute exacerbation chronic obstructive pulmonary disease (AECOPD). In this study, T-IgE in AECOPD patients were investigated and jointly analyzed with the clinical characteristics. AECOPD patients hospitalized from July 2018 to July 2019 were included in this study. In this patient cohort, clinical information was investigated. Routine blood tests, C-reactive protein and T-IgE levels of patients were determined along with blood gas analysis. The length of hospital stays, mechanical ventilation during hospitalization, ICU admission, glucocorticoid related clinical information were recorded. A total of 285 AECOPD patients were included in this study, which consisted of a high proportion of males. Of all patients, 49.82% patients exhibited higher T-IgE levels. Based on the reference T-IgE value 60 kU/L, patients were divided into high T-IgE group with T-IgE > 60 kU/L, and low T-IgE group with T-IgE ≤ 60 kU/L. There was no significant difference in the dosage of glucocorticoid between the two groups. Patients in the high T-IgE group had shorter hospital stays and lower probability of mechanical ventilation compared to the low T-IgE group. After adjustment for confounding factors, T-IgE was negatively correlated with the length of hospital stays. AECOPD patients with elevated T-IgE had shorter hospital stays and lower risks of mechanical ventilation and ICU admission. Our results showed that T-IgE might play an important role on evaluating the condition and guiding for treatment decisions in AECOPD patients.

## 1. Introduction

Chronic obstructive pulmonary disease (COPD) is a common and frequently occurring lung disease that is a serious threat to human health. It has been indicated that the prevalence of COPD worldwide is 10.1%^[1]^ and 13.7%^[2]^ in the Chinese population over 40 years of age. Acute exacerbation chronic obstructive pulmonary disease (AECOPD) is a process of exacerbation of respiratory symptoms and acute onset of chronic disease in COPD patients. The typical manifestations of AECOPD include exacerbated dyspnea, aggravated cough, increased sputum volume and/or purulent sputum beyond daytime variation. Acute exacerbation is an essential event in the progression of COPD, which seriously affects patient quality of life and increases readmission rates and mortality.^[3,4]^ AECOPD is a heterogeneous condition and its diagnosis is nonspecific and dependent on subjective assessment by clinicians. Currently, the treatment of patients with different phenotypes of AECOPD is mainly based on clinician experience and lacks specific treatments.^[3]^ Therefore, further research is urgently required to identify clinical biomarkers for AECOPD patients, thereby indicating disease-specific phenotypes and informing treatment decisions in the clinic.

Allergic reactions have been proposed as common factors in asthma and COPD patients. Currently, the role of allergy in the pathogenesis of asthma has been generally recognized but its role in COPD, especially in AECOPD, has rarely been extensively studied.^[5]^ It has been suggested that the allergic phenotype is a particular subtype of COPD, and COPD patients with allergic symptoms have severe respiratory symptoms and frequent exacerbation history.^[6]^ Immunoglobulin E (IgE) is an antibody that mediates allergic reactions and has important value in the diagnosis of various allergic diseases.^[7]^ IgE-related allergic diseases have been shown to affect about 30% of the population worldwide.^[8]^ A longitudinal study of COPD has shown that total serum IgE (T-IgE) levels are negatively correlated with FEV1 (forced expiratory volume in one second)/FVC (forced vital capacity).^[9]^ In another study, T-IgE levels are associated with impaired lung function impairment and respiratory symptoms in patients with COPD (e.g., dyspnea).^[10]^ Currently, few studies have demonstrated the relationship between IgE and AECOPD. Also, the utility of T-IgE levels as a biomarker to guide AECOPD treatments remains to be further investigated.

This study aimed to investigate the T-IgE levels in the serum of AECOPD patients and to evaluate its clinical characteristics of the allergic phenotype. Furthermore, we also evaluated the potential of T-IgE as a biomarker in the clinical management of AECOPD.

## 2. Materials and methods

### 2.1. Study subjects and ethic statement

Our present work retrospectively studied the AECOPD patients that were hospitalized at the Liaocheng People’s Hospital from July 2018 to July 2019 (Shandong, China). This study was approved by the Ethical Committee of Liaocheng People’s Hospital [approval no. 2021078], in accordance with the declaration of Helsinki. The informed consents were obtained from all participants.

During the hospitalization, all patient treatments were decided by the physician-in-charge and were not affected by our study, including but not limited to anti-infection treatments, systemic glucocorticoid administration, adjuvant treatments (e.g. oxygen therapy or mechanical ventilation), discharge, etc.

### 2.2. Inclusion and exclusion criteria

The inclusion criteria for the study were summarized as below:

1. patients over 40 years old with risk factors (including smoking) for COPD;
2. patients that undergone pulmonary function tests at the stable stage (at least one month before admission) and met the criteria of FEV1/FVC < 0.7 after bronchodilator inhalation;
3. patients with acute symptoms on admission, such as acute exacerbation of respiratory symptoms beyond daytime variation requiring medication adjustment.

The following patients were excluded from our study:

1. patients with allergic rhinitis and other allergic diseases;
2. patients with bronchial asthma (including asthma-COPD overlap (ACO)), allergic bronchopulmonary aspergillosis, pneumonia, bronchiectasis, interstitial lung disease and other respiratory diseases;
3. patients with acute left heart failure and other unstable heart diseases;
4. patients who died during discharge without doctor’s advice or hospitalization;
5. patients without complete T-IgE or other related laboratory tests.

### 2.3. Clinical features of all samples

Clinical information of all samples were collected, included demographic variables, body mass index (BMI), smoking history, lung function (after inhalation of bronchodilator), the frequency of acute exacerbation episodes during the past 12 months, the course of COPD, medication history (regular glucocorticoid inhalation) and complications (ischemic cardiomyopathy, hypertension, cardiac insufficiency and diabetes). Moreover, routine blood tests, C-reactive protein, serum T-IgE levels and blood gas analysis were conducted before treatment. The serum T-IgE levels were measured in Liaocheng Key Laboratory of Respiratory Diseases (ImmunoCap TM 100, Pharmacia Company, Sweden). According to the reference value 60 kU/L of the instrument, patients with serum T-IgE levels > 60 kU/L were abnormal. Other tests were performed by the clinical laboratory of the Liaocheng People’s hospital. Meanwhile, the length of hospital stay, mechanical ventilation during hospitalization (including noninvasive and invasive mechanical ventilation), ICU admission, glucocorticoid administration, cumulative dose of glucocorticoid administration during hospitalization, days of glucocorticoid administration and average dosage glucocorticoid (cumulative dose/administration days) information were recorded. The dose of glucocorticoid was converted to the dose of methylprednisolone.

### 2.4. Statistical analysis

The categorical variables were presented as numbers and percentages, and the continuous variables were presented as the mean ± standard deviation. A chi-square test was used to determine the significance of difference between two groups. A Kolmogorov-Smirnov test was used to determine the difference significance of the normally distributed data. A Two-sample *t* test or Mann–Whitney *U* test was used for 2 sample continuous variables with a normal and non-normal distribution. Binary Logistic regression and multiple linear regression analysis were used to evaluate the relationship between dependent and independent variables. All data were statistically analyzed using IBM SPSS 25.0 (IBM Corphan, Armonk, NY).

## 3. Results

### 3.1. The clinical features between high and low T-IgE AECOPD patients

**Figure 1.**
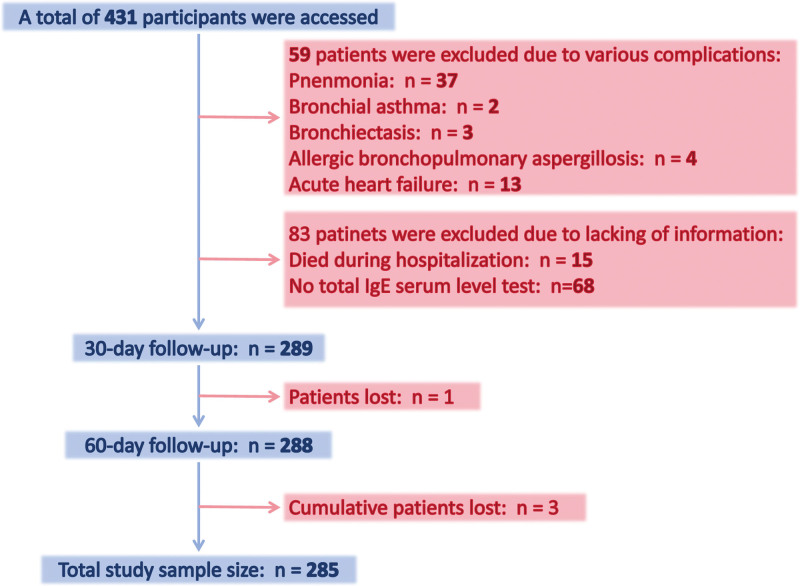
The CONSORT figure of this present work.

A total of 431 AECOPD patients were included in this study. Patients with complications including 37 cases of pneumonia, 2 cases of bronchial asthma, 3 cases of bronchiectasis, 4 cases of allergic bronchopulmonary aspergillosis and 13 cases of acute left heart failure were excluded from the analysis. 15 patients died during hospitalization, 68 cases did not have total IgE serum level tests and 4 cases were automatically discharged. The detailed sample exclusion was displayed in Figure 1, and 285 patients were finally included in our subsequent analysis.

Next, based on the reference T-IgE value 60 kU/L, all 285 AECOPD patients were divided into high T-IgE group with T-IgE > 60 kU/L (n = 142), and low T-IgE group with T-IgE ≤ 60 kU/L (n = 143). We found that the serum T-IgE data was not normally distributed (statistical K-S = 0.286, *P* < .01), thus the serum T-IgE was converted using logarithmic transformation method.^[11]^ The logarithmically transformed T-IgE (ln-T-IgE) values were in accordance with normal distribution (statistical K-S = 0.040, *P* = .200). Among all patients, 49.82% patients exhibited elevated T-IgE levels. The clinical characteristics of high and low T-IgE AECOPD patients were summarized in Table 1.

**Table 1:** Baseline characteristics of high T-IgE and low T-IgE in patients.

Variables	Total patients (n = 285)	high T-IgE (n = 142)	low T-IgE (n = 143)	*P* value
Age (yr)	70.75 ± 8.56	70.11 ± 8.63	71.38 ± 8.47	.212*
Male	211 (74.1)	117 (41.1)	94 (33.0)	**.001** ‡
BMI (kg/m^2^)	22.20 ± 4.57	21.57 ± 4.29	22.83 ± 4.77	**.019** *
Smoking status				
Current smokers	80 (28.1 )	45 (15.8)	35 (12.3)	.087‡
Ex-smokers	118 (41.4)	62 (21.8)	56 (19.6)	
No-smokers	87 (30.5)	35 (12.3)	52 (18.2)	
Duration of COPD (yr)	9.62 ± 7.00	8.99 ± 10.79	10.24 ± 11.34	.194†
Number of exacerbation in the past 12 mo	1.37 ± 1.00	1.37 ± 1.78	1.37 ± 1.05	.696†
Spirometric value (post-bronchodilator)				
FEV1(L)	0.87 ± 0.23	0.86 ± 0.25	0.88 ± 0.22	.586*
FVC(L)	1.96 ± 1.26	1.89 ± 0.39	2.04 ± 1.73	.335*
FEV1/FVC ratio (%)	0.46 ± 0.08	0.46 ± 0.08	0.46 ± 0.09	.627*
ICS use	150 (52.6)	89 (31.2)	61 (21.4)	**.001** ‡
Co-morbidity				
Hypertension	96 (33.7)	4 (1.4)	92 (32.3)	**<.001** ‡
Diabetes mellitus	35 (12.3)	7 (2.5)	28 (9.8)	**<.001** ‡
Heart failure	21 (7.4)	7 (2.5)	14 (4.9)	.116‡
Ischaemic heart disease	107 (37.5)	44 (15.4)	63 (22.1)	**.023** ‡

Bold values are statistically significant (*P* < 0.05).

Data are expressed as mean ± SD, n (%).

BMI = body mass index, CAT = COPD assessment test, CCQ = Clinical COPD Questionnaire, FEV1 = forced expiratory volume in one second, FVC = forced vital capacity, ICS = inhaled corticosteroid, mMRC = modified Medical research Council.

* *P* values were calculated by Student’s *t* test.

† *P* values were calculated by the Mann–Whitney *U* test.

‡ *P* values were calculated by the chi-square test.

### 3.2. The laboratory test results of high and low T-IgE AECOPD patients

The average age of the patients was 70.75 ± 8.56 years and there was no significant difference in age between the two groups (*P* > .05). 74.1% of the patients were male and the proportion of males in the high T-IgE group was higher than that in the low T-IgE group (*P* < .05). Compared to the low T-IgE group, the proportion of patients with glucocorticoid inhalation was higher in the high T-IgE group, whilst the proportion of BMI and complications (hypertension, diabetes and ischemic heart disease) was significantly lower (*P* < .05). There was no significant difference in the proportion of smokers, the frequency of acute exacerbation episodes in the past 12 months, the course of COPD, pulmonary function parameters and the proportion of heart failure complication between the two groups (*P* > .05). The laboratory parameters of the two groups are summarized in Table 2. The laboratory test results after admission showed that the pH value in the high T-IgE group was higher than that in low T-IgE group (*P* < .05). PaCO2 was lower in the high T-IgE group compared to the low T-IgE group (*P* < .05). There were no significant differences in white blood cell count and the levels of neutrophils, eosinophils, CRP and PaO2 between the two groups (*P* > .05).

**Table 2:** Laboratory variables of the patients (n = 285).

Variables	Total patients (n = 285)	high T-IgE (n = 142)	low T-IgE (n = 143)	*P* value
T-IgE (kU/L)	140.33 ± 167.58	257.04 ± 170.10	24.43 ± 16.98	**<.001** †
Ln (T-IgE)	1.78 ± 0.63	2.32 ± 0.28	1.25 ± 0.38	**<.001** *
Leucocyte count (10^9^L)	8.90 ± 4.33	8.97 ± 3.72	8.83 ± 4.88	.784*
Neutrophil count (10^9^L)	7.32 ± 6.34	7.57 ± 7.05	7.06 ± 5.55	.499*
Eosinophilic count (10^9^L)	0.14 ± 0.62	0.12 ± 0.24	0.17 ± 0.84	.484*
CRP (mg/dL)	36.02 ± 43.95	39.49 ± 52.77	32.57 ± 30.01	.667†
pH	7.40 ± 0.07	7.41 ± 0.06	7.39 ± 0.07	**.008** *
PaO_2_ (mm Hg)	78.59 ± 26.11	77.55 ± 25.57	79.62 ± 26.69	.506*
PaCO_2_ (mm Hg)	50.91 ± 16.54	48.96 ± 14.61	52.84 ± 18.11	**.048** *

Data are expressed as mean ± SD.

CRP = C-reactive protein, PaCO_2_ = arterial carbon dioxide partial pressure, PaO_2_ = arterial oxygen partial pressure, T-IgE = serum total IgE.

* *P* values were calculated by Student’s *t* test.

† *P* values were calculated by the Mann–Whitney *U* test.

### 3.3. Results of joint analysis of all clinical features in high and low T-IgE AECOPD patients

Subsequently, the clinical features were then jointly analyzed in high and low T-IgE AECOPD patients. 32.3% of patients in the high T-IgE group and 34% of patients in the low T-IgE group received systemic glucocorticoid treatment. Clinical variables were summarized in Table 3. No significant differences were observed in the dosage of glucocorticoid during the treatment (including the number of patients treating with glucocorticoid, the average dosage of glucocorticoid, cumulative dosage and medication time) between the two groups (*P* > .05).

**Table 3:** Clinical variables of the patients (n = 285).

Variables	High T-IgE (n = 142)	Low T-IgE (n = 143)	*P* value
No of using Systemic Corticosteroids of patients	92 (32.3)	97 (34.0)	.587†
Average daily does of Systemic Corticosteroids (mg)	26.84 ± 27.54	30.11 ± 35.54	.539*
Average cumulative does of Systemic Corticosteroids (mg)	203.32 ± 234.88	180.06 ± 179.50	.699*
Length of systemic corticosteroids using (d)	5.83 ± 5.20	5.24 ± 4.80	.428*
Length of hospital stay, (d)	9.49 ± 3.05	10.59 ± 3.42	**.004** *
ICU admission	11 (3.9)	29 (10.2)	**.002** †
Mechanical ventilation	15 (5.3)	41 (14.4)	**.000** †

Data are expressed as n (%) or median (1st quartile; 3rd quartile).

* *P* values were calculated by the Mann–Whitney *U* test.

† *P* values were calculated by the chi-square test.

Compared to patients in the low T-IgE group, patients in the high T-IgE group had shorter hospital stays (9.49 ± 3.05 vs 10.59 ± 3.42, *P* < .01) and lower probabilities of ICU admission (3.9% vs 10.2%, *P* < .01) and mechanical ventilation (5.3% vs 14.4%, *P* < .01) (Table 3). As ln-T-IgE was normally distributed after logarithmic transformation, linear regression was applied to analyze the relationships between the ln-T-IgE and related variables. We found that males (β = −0.147, 95% CI: −0.234 to −0.060, *P* < .01), hypertension (β = −0.970, 95% CI: −1.055 to −0.885, *P* < .01), diabetes (β = −0.357, 95% CI: −0.474 to −0.239, *P* < .01), PaCO2 (β = −0.003, 95% CI: −0.006 to −0.000, *P* < .01) and the length of the hospital stay (β = −0.017, 95% CI: −0.029 to −0.006, *P* < .01) were negatively correlated to the ln-T-IgE. The application of inhaled corticosteroid was related to ln-T-IgE (β = 0.409, 95% CI: 0.331–0.478, *P* < .01). BMI, ischemic heart disease and PH values had no significant effect on ln-T-IgE (Table 4). Univariate Logistic regression analysis showed that low T-IgE levels, ischemic heart disease, high BMI, high PaCO2 and low pH were risk factors for mechanical ventilation and ICU admission in COPD patients. Multivariate Logistic regression analysis showed that after correction for ischemic heart disease, BMI, PaCO2 and pH value, T-IgE was still a significant risk factor for mechanical ventilation and ICU admission in COPD patients (Tables 5 and 6).

**Table 4:** Multivariable linear regression analysis of Ln (T-IgE) and variables (n = 285).

Variables	β	Std.Error	95% CI	*P* value
Male	−0.147	0.044	−0.234 to −0.060	.001
BMI (kg/m ^2^)	−0.008	0.004	−0.016 to 0.000	.060
ICS use	0.409	0.039	0.331 to 0.478	**<.001**
Hypertension	−0.970	0.043	−1.055 to −0.885	<.001
Diabetes mellitus)	−0.357	0.060	−0.474to −0.239	<.001
Ischaemic heart disease	−0.002	0.041	−0.083 to 0.082	.962
pH	−0.061	0.352	−0.755 to 0.632	.862
paCO2 (mm Hg)	−0.003	0.001	−0.006 to −0.000	.032
Length of hospital stay (d)	−0.017	0.006	−0.029 to −0.006	**.003**

BMI = body mass index, CI = confidence interval, ICS = inhaled corticosteroid, PaCO_2_ = arterial carbon dioxide partial pressure, PaO_2_ = arterial oxygen partial pressure, T-IgE = serum total IgE.

**Table 5:** Results of univariable and multivariate analysis of correlation between baseline characteristics, laboratory test results and mechanical ventilation events.

Variables	Univariable analysis			Multivariate analysis		
	OR	95% CI	*P* value	OR	95% CI	*P* value
T-IgE	0.995	0.992–0.998	0.001	0.996	0.993–0.999	0.005
Sex						
Male	1					
Female	1.625	0.865–3.053	.132			
Smoking status						
Never smoker	1					
Current smoker	0.610	0.282–1.318	.208			
Former smoker	0.720	0.367–1.415	.341			
Drug						
No	1					
ICS	0.560	0.309–1.013	.055			
Co-morbidities						
Ischemic heart disease	3.005	1.649–5.477	**<.001**	2.872	1.500–5.499	**.001**
Cardiac insufficiency	0.409	0.092–1.812	.239			
Hypertension	1.603	0.882–2.914	.122			
Diabetes mellitus	1.774	0.797–3.984	.160			
Age	1.019	0.984–1.055	.294			
BMI	1.065	1.000–1.134	**.050**	1.035	0.968–1.106	.317
Disease duration(year)	0.988	0.960–1.017	.416			
Acute exacerbations of COPD	0.845	0.637–1.119	.240			
FEV1 (L)	1.400	0.421–4.656	.584			
FVC (L)	0.831	0.420–1.644	.595			
FEV1/FVC (%)	8.425	0.334–212.723	.196			
WBC (×10^9^/L)	1.026	0.962–1.093	.493			
Neutrophil (×10^9^/L)	0.984	0.928–1.043	.580			
Eosinophil (×10^9^/L)	1.353	0.831–2.204	.225			
CRP	1.003	0.997–1.009	.340			
PH	0.015	0.000–0.720	**.034**	1.529	0.008–299.844	.875
PO_2_	1.006	0.995–1.017	.266			
PCO_2_	1.019	1.002–1.036	**.024**	1.019	0.998–1.040	.075

BMI = body mass index, CAT = COPD assessment test, CCQ = Clinical COPD Questionnaire, CI = confidence interval, CRP = C-reactive protein, FEV1 = forced expiratory volume in one second, FVC = forced vital capacity, ICS = inhaled corticosteroid, mMRC = modified Medical research Council, OR = odds ratio, PaCO2 = arterial carbon dioxide partial pressure, PaO2 = arterial oxygen partial pressure, T-IgE = serum total IgE.

**Table 6:** Results of univariable and multivariate analysis of correlation between baseline characteristics, laboratory test results and ICU admission.

Variables	Univariable analysis			Multivariate analysis		
	OR	95% CI	*P* value	OR	95% CI	*P* vale
T-IgE	0.994	0.990–0.998	**.003**	0.995	0.992–0.999	**.016**
Sex						
Male	1					
Female	0.913	0.431–1.934	.811			
Smoking status						
Never smoker	1					
Current smoker	0.951	0.428–2.109	.901			
Former smoker	0.947	0.418–2.146	.896			
Drug						
No	1					
ICS	1.811	0.917–3.577	.087			
Co-morbidities						
Ischemic heart disease	3.313	1.657–6.625	**.001**	3.253	1.518–6.970	**.02**
Cardiac insufficiency	0.288	0.038–2.212	.232			
Hypertension	1.524	0.771–3.013	.225			
Diabetes mellitus	2.019	0.844–4.827	.114			
Age	1.033	0.992–1.076	.120			
BMI	1.101	1.025–1.183	**.008**	1.072	0.994–1.157	.071
Disease duration (yr)	0.975	0.940–1.012	.182			
Acute exacerbations of COPD	0.770	0.548–1.082	.132			
FEV1 (L)	1.088	0.264–4.418	.907			
FVC (L)	0.897	0.505–1.592	.710			
FEV1/FVC (%)	1.788	0.037–86.553	.769			
WBC(×10^9^/L)	1.035	0.965–1.110	.340			
Neutrophil (×10^9^/L)	0.976	0.904–1.053	.525			
Eosinophil (×10^9^/L)	1.467	0.842–2.555	.176			
CRP	1.004	0.998–1.011	.190			
PH	0.014	0.000–0.907	**.045**	11.809	0.031–445.012	.416
PO_2_	1.003	0.990–1.015	.658			
PCO_2_	1.025	1.007–1.043	**.007**	1.030	1.006–1.053	**.012**

BMI = body mass index, CAT = COPD assessment test, CCQ = Clinical COPD Questionnaire, CRP = C-reactive protein, CI = confidence interval, FEV1 = forced expiratory volume in one second, FVC = forced vital capacity, ICS = inhaled corticosteroid, mMRC = modified Medical research Council, OR = odds ratio, PaCO_2_ = arterial carbon dioxide partial pressure, PaO_2_ = arterial oxygen partial pressure, T-IgE = serum total IgE.

## 4. Discussion

COPD has been established as a complicated heterogenous condition involving multiple pathological processes, and acute exacerbations of COPD often result in symptomatic deterioration, lung injury, and higher death risk.^[12]^ Previous research into the allergic phenotype and COPD has mainly focused on the stable phase. Studies have emphasized the relationship between the allergic phenotype and the epidemiological characteristics of COPD patients, clinical symptoms and lung function. Jamieson et al found that 25% to 30% of COPD patients manifest an allergic phenotype.^[6]^ Moreover, another study that investigated T-IgE as an allergy biomarker showed that 47.3% of COPD patients had elevated serum levels of total IgE.^[10]^ A recently published META analysis showed that about one-third of COPD patients have allergic symptoms.^[13]^ Nevertheless, in our present work, we have focused on the T-IgE in AECOPD patients.

The allergic symptoms in COPD patients have been shown to correlate with occupation, dust exposure and other factors. These are accompanied by obvious respiratory symptoms, poor lung function and frequent acute exacerbation.^[6,10,14–16]^ These studies have suggested that the allergic phenotype may be important phenotypes in COPD, while it was limited to the stable stage of disease and they did not assess COPD patients with acute exacerbation. AECOPD has a variety of clinical manifestations and pathophysiological characteristics, thus AECOPD has been divided into various phenotypes based on heterogeneity. AECOPD patients were indicated to be divided in four subtypes: bacterial predominant, viral predominant, eosinophilic predominant and pauci-inflammatory subtypes.^[17]^ Arostegui et al have classified AECOPD into four subtypes (subtype A–D) according to the severity of clinical symptoms and complications.^[18]^ Gulati et al^[19]^ classified patients into 4 subtypes (E1–E4) according to pathological and clinical characteristics. While, the current classification of AECOPD phenotype did not fully account for the clinical characteristics of AECOPD. In this study, we showed that many AECOPD patients have elevated T-IgE levels and the allergic phenotype may be particularly important in the clinic.

Systemic glucocorticoid treatment is an important therapy for AECOPD that can reduce the rates of treatment failure and rehospitalization in patients with non-respiratory failure.^[20]^ Although the recommended dose of systemic glucocorticoid have been proposed in the GOLD guidelines, differences in the dose and course of treatment remain in the clinic.^[4,21]^ In our present study, we did not find a significant correlation between T-IgE and dose of systemic glucocorticoid in AECOPD, while it was still an important aspect in our further work, considering the vital role of the treatment.

The prolonged hospitalization of AECOPD patients is an important factor that contributes to increased medical expenses and personal burden. The early identification of COPD patients with acute exacerbation can reduce the occurrence of adverse events and treatment costs, as AECOPD patients often needed long-term hospitalization. Current studies have found that age, disease severity, complications and acute respiratory acidosis are important predictors of prolonged hospitalization in AECOPD patients.^[22,23]^ Our study suggested that T-IgE levels were promising in predicting hospitalization in AECOPD patients. Whereas, considering the limited sample size of our work, further reliable predictive value of T-IgE level in long-term hospitalization of AECOPD patients deserved to be explored in an expanded population.

Moreover, mechanical ventilation is an important treatment for AECOPD patients with respiratory failure that can reduce mortality during hospitalization.^[21]^ Currently, there has been no consensus standard for AECOPD patients to be admitted to ICU administration that can importantly inform clinical decision-making. Moreover, lack of reliable biomarkers greatly limited in guiding clinicians in identifying patients for ICU admission.^[24]^ Compared to the control group, patients with elevated IgE levels had a lower risk of mechanical ventilation and ICU admission. These data suggested that total serum IgE levels could be a useful biomarker to assess the severity of AECOPD and inform decisions for patients requiring mechanical ventilation treatment and ICU admission. Our data implied that T-IgE levels might play an important role in evaluating the condition of AECOPD patients and guiding treatment decisions.

Currently, the underlying mechanism supporting our observations remained largely unknown. Previous studies have shown that IgE is one of the most important initiators of allergic inflammation.^[25]^ It has been suggested that the IgE level was associated with pulmonary inflammation and airway remodeling in patients with stable COPD. Elevated IgE levels may aggravate lung inflammation and promote airway remodeling in COPD patients resulting in decreased lung function and severe respiratory symptoms.^[6,10]^ Exogenous IgE could bind to the FceR I receptor on the surface of effector cells and exert biological effects including the initiation and maintenance of airway inflammation, thereby inducing airway hyper responsiveness.^[7,26,27]^ Glucocorticoids have been shown to exhibit anti-inflammatory effects.^[28]^ Bronchodilators, glucocorticoids and other drugs used in treatment could alleviate the hyperresponsiveness of airways during the acute exacerbation phase and inhibit the related inflammatory response.^[29]^ Consequently, the hospitalization time and the risk of mechanical ventilation in AECOPD patients could be reduced. However, bronchodilators and glucocorticoids do not directly affect the T-IgE levels nor do they guide the dosage of glucocorticoids.

Previous studies of IgE and COPD have focused on stable COPD. Our study has explored the relationship between T-IgE levels and the phenotype of AECOPD. We have demonstrated a significant potential for IgE as a biomarker in the diagnosis, treatment and classification of AECOPD. However, there were several limitations in this study. Our work was mainly based on retrospective analysis of a relative small sample size, which might partly limit our findings. Additionally, owing to the short follow-up time, the impacts of some confounding factors (like seasonal factors) on AECOPD patients have not been well excluded. Integrating the above limitations, our findings deserved to be further validated in an expanded sample size, which would be more conducive to the clinical application of our results.

## 5. Conclusions

To summarize, in this work, the crucial correlation between T-IgE level and AECOPD as well as the related clinical features has been jointly investigated. In AECOPD patients, elevated T-IgE levels are observed, which might be connected with the allergic phenotype of AECOPD patients. Meanwhile, T-IgE levels are significantly associated with the hospital stay, risk of mechanical ventilation, and ICU admission. Our findings provide more insights into the potential correlation between elevated T-IgE level and AECOPD onset, which is expected to benefit for the evaluation of AECOPD condition and clinical management.

## Acknowledgments

The authors thank all patients for their participation. We thank Zhen Li, Jun Qi, Lili Li, and Tingting Jiang Tian Xiufen for assistance in patient recruitment. We thank Juan Zheng for performing the serum total IgE measurements, and we thank all technicians of the laboratory for Clinical Chemistry (the Liaocheng People’s hospital) for assistance with sample preparation, measurements, and data collection. We thank Wang Yan, Peige Zhao and Lindong Yuan for their expert support in the planning of this study protocol.

## Author contributions

**Conceptualization:** Peige Zhao.

**Data curation:** Xiang Xie, Lindong Yuan.

**Funding acquisition:** Xiang Xie.

**Investigation:** Xiang Xie, Lili Li.

**Methodology:** Xiang Xie, Zhen Li.

**Project administration:** Xiang Xie, Peige Zhao.

**Resources:** Tingting Jiang.

**Software:** Jun Qi, Shan Qin.

**Supervision:** Xiufen Tian.

**Validation:** Xiang Xie, Juan Zheng.

**Writing – original draft:** Xiang Xie, Ziyun Yang, Yan Wang, Peige Zhao.

**Writing – review & editing:** Xiang Xie, Ziyun Yang, Yan Wang, Peige Zhao.
